# Neuromuscular Activation Strategies of the Lower Limb During Maximal Sprinting in Youth Track and Field Athletes: Age-Related Differences and Implications for Talent Identification

**DOI:** 10.3390/sports14080353

**Published:** 2026-08-17

**Authors:** Gaku Kakehata, Tuncay Örs, Sofyan Sahrom, Chee Yong Low

**Affiliations:** 1 Faculty of Sport Sciences, Waseda University, 2-579-15 Mikajima, Tokorozawa 359-1192, Saitama, Japan; 2 High Performance Sport Institute, 3 Stadium Drive, Singapore 397630, Singapore; tuncay_ors@hpsi.spexsg.org (T.Ö.); sofyan_sahrom@hpsi.spexsg.org (S.S.); low_cheeyong@hpsi.spexsg.org (C.Y.L.)

**Keywords:** electromyography, sprint running, youth athletes, rectus femoris, neuromuscular development, motor unit recruitment, talent identification

## Abstract

Sprint performance improves throughout adolescence as a result of both structural and neuromuscular development. In particular, increases in body height, lower limb length, and muscle volume have been consistently linked to improvements in sprint performance. However, allometric scaling of force, speed, and power remain lower in adolescents compared to adults even after structural differences are accounted for, implicating neural factors as independent contributors to performance development. The purpose of this study was to investigate differences in neuromuscular activation patterns of the lower limb muscles during maximal sprinting between youth male athletes across two age groups (U19: 17–19 years; U16: 13–16 years). Eighteen athletes performed a 50 m maximal sprint. Spatiotemporal variables (running speed, step frequency, step length) were measured over 30–50 m using a high-speed camera (240 Hz) and timing gates. Electromyographic (EMG) signals were recorded simultaneously from ten lower limb muscles using wireless EMG sensors (2000 Hz): rectus femoris (RF), biceps femoris (BF), semitendinosus (ST), gluteus maximus (Gmax), gluteus medius (Gmed), vastus lateralis (VL), vastus medialis (VM), tibialis anterior (TA), gastrocnemius (GAS), and soleus (SOL). Root mean square (RMS) amplitude was calculated across four gait phases (contact, early-swing, mid-swing, late-swing) and normalised to maximal voluntary Isometric contraction (%MVIC). The U19 group demonstrated significantly greater running speed (U19: 9.49 ± 0.39 vs. U16: 8.67 ± 0.25 m·s^−1^, *p* < 0.001), step frequency (U19: 4.49 ± 0.12 vs. U16: 4.35 ± 0.16 Hz, *p* = 0.004), and step length (U19: 2.12 ± 0.12 vs. U16: 1.99 ± 0.06 m, *p* = 0.010) than U16. The overall pattern of lower limb muscle activation across the gait cycle was broadly similar between groups; however, a significant group × phase interaction was observed for RF (*p* = 0.003, *F* = 5.257, *η*^2^ = 0.247), with post hoc analysis revealing greater RF activation during early swing in U19 (*p* = 0.033). These findings may indicate that sprint-specific training in youth athletes is associated with not only structural but also neuromuscular differences, specifically reflecting enhanced RF recruitment during the phase-critical moment of early swing—a window in which high-threshold motor unit activation is most mechanically decisive. EMG-based assessment of hip flexor activation during maximal sprinting may provide a complementary tool, pending further validation, for talent identification and training prescription in youth track and field.

## 1. Introduction

Sprint performance is a fundamental athletic capacity that underpins success across a wide range of sports, and its development during childhood and adolescence has long been a focus of sports science research [1,2]. The adolescent period (approximately 10–19 years of age, per the WHO classification) represents a dynamic phase of structural development, encompassing rapid increases in body height, lower limb length, muscle volume, and bone mass that collectively reshape the mechanical basis of athletic performance [3]. These morphological changes are well-documented determinants of sprint ability: greater muscle size and longer limbs directly contribute to increased step length and ground reaction force production during running [4,5]. Maximum sprinting speed is therefore traditionally understood as the product of step length and step frequency [6,7], and the improvement in both quantities across the adolescent years is in large part attributed to these ongoing structural gains.

However, growing evidence indicates that structural development alone is insufficient to explain the full extent of performance differences observed between youth athletes and adults, or across different age groups within youth athletic populations. When force, speed, and power are scaled allometrically to account for body size, these measures remain lower in adolescents than in adults, suggesting that neural factors are independent contributors to performance development [8]. Importantly, it should be noted that chronological age and biological maturation are distinct constructs. During adolescence, substantial inter-individual variability in maturation status means that athletes of the same chronological age may differ markedly in their neuromuscular developmental stage [5,9]. Biological maturation—typically quantified via maturity offset—may substantially influence neuromuscular characteristics and sprint performance independently of chronological age. This variability must be considered when interpreting age-group differences in neuromuscular function. It has been hypothesised that performance gaps between younger and older athletes are attributable, at least in part, to a lesser ability in less mature athletes to recruit and fully utilise high-threshold, type II motor units—the fast-twitch fibres most relevant to explosive, high-velocity movements such as sprinting [8,10]. Supporting this interpretation, the neural mechanisms underpinning force production—including differential motor unit recruitment, muscle pre-activation, agonist–antagonist co-contraction, and stretch reflex control—all undergo substantial maturation during adolescence and develop independently of structural changes in muscle size [8,11].

Consistent with this dual structural–neural framework, marked age-related differences in sprint performance emerge most prominently during mid-adolescence [5,9]. Prior to peak height velocity (PHV), improvements in sprinting performance are primarily attributed to central nervous system adaptations, including enhanced neuromuscular recruitment and inter-muscular coordination, whereas post-pubertal gains are substantially driven by the additional influence of anabolic hormones on muscle mass, cross-sectional area, and anaerobic metabolic capacity [9,12]. Despite this, the precise nature of neuromuscular adaptations during sprinting across different adolescent age groups remains poorly characterised. Indeed, muscle–tendon unit behaviour during sprinting has been shown to differ between adolescent and adult sprinters in a manner not fully accounted for by structural differences alone [13].

The thigh muscles play a central role in the generation and control of sprinting kinematics, particularly during the swing phase. Electromyographic (EMG) studies have demonstrated that the lower limb muscles exhibit highly coordinated, phase-specific activation patterns during sprinting [14,15,16]. Among these, the rectus femoris (RF)—a biarticular muscle spanning both the hip and knee joints—exhibits a distinctive two-peak activation pattern: one peak during mid-stance and a second, biomechanically significant peak during the swing phase [16]. During early swing, the primary functional role of RF is hip flexion, which drives rapid forward leg recovery and contributes to higher step frequency [16]. Previous research in adult sprinters has shown that higher step frequency is associated with earlier timing of RF activation relative to antagonist biceps femoris (BF) activity [16]. Furthermore, meta-analytic evidence has confirmed that increased sprint speed is associated with greater activation of the posterior thigh muscles and gluteus maximus [15].

Despite this growing understanding of sprint neuromechanics, relatively little is known about how neuromuscular activation strategies during sprinting differ across age groups within youth athletic populations. Talent identification in youth sprinters has traditionally been guided by anthropometric measures and field-based performance tests such as sprint time, jump height, and strength scores [17,18]. Contemporary talent identification frameworks, however, are widely recognised as multidimensional, integrating anthropometric, physical, technical, psychological, and maturational characteristics [17]. Within this multidimensional context, neuromuscular activation assessed via surface EMG may offer a complementary dimension of information, although its practical application for talent identification under field conditions requires further validation, as surface EMG primarily reflects muscle activation patterns rather than directly quantifying motor unit recruitment or confirming specific neural adaptations. Meaningful improvements in muscle size, pennation angle, fascicle length, and tendon stiffness occur with growth and maturation [12], and resistance training in youth athletes can further augment motor unit recruitment and reduce electromechanical delay [19]. Nevertheless, whether sport-specific sprint training in youth athletes leads to age-related differences in the magnitude of muscle activation during maximal sprinting—and whether such differences may reflect neuromuscular adaptations beyond structural growth—has yet to be systematically examined.

Recent work has begun to address this gap. Phase-specific agonist–antagonist coordination of the rectus femoris and biceps femoris has been compared between adults and adolescents during 50 m sprinting, with adolescents showing greater co-contraction during the contact and propulsive phases, but no significant group differences in any swing-related phase [20]. That study, however, contrasted adults with a single adolescent group and quantified coordination within one biarticular muscle pair. The magnitude of phase-specific activation across the wider lower-limb musculature, and differences between age groups within a youth athlete population, therefore remain uncharacterised.

This gap is significant from both a scientific and practical standpoint. Electromyography provides a quantitative assessment of muscle activation patterns that cannot be inferred from kinematic or kinetic measurements alone [21,22]. If neuromuscular activation strategies during maximal sprint running differ between age groups within youth athletics, such findings may suggest that sprint-specific training is associated with neural differences—such as differences in motor unit recruitment and inter-muscular coordination—although a cross-sectional design cannot confirm this interpretation causally. Identifying these neuromuscular differences would provide a scientific basis for expanding talent identification frameworks beyond conventional structural assessments. To date, no study has employed multi-channel EMG to compare lower-limb neuromuscular activation patterns across different age groups of youth track and field athletes during maximal sprint running. To our knowledge, no study has employed multi-channel EMG across the major lower-limb muscle groups to compare phase-specific activation amplitude between age groups within a trained youth track and field population during maximal sprint running.

Therefore, the purpose of this study was to investigate differences in neuromuscular activation patterns of the lower limb muscles during maximal sprinting between youth athletes of different age groups. We hypothesised that if neuromuscular activation patterns differ between age groups, this may reflect age-related differences in neural control strategies—potentially including motor unit recruitment—that are associated with accumulated sprint training and maturation. However, given the cross-sectional design, findings should be interpreted as associations rather than evidence of causal neuromuscular adaptation. Such findings would have direct implications for sport-specific testing, athlete screening, and talent development strategies for youth sprinters.

## 2. Materials and Methods

### 2.1. Participants

Eighteen male youth track and field athletes participated in this study. Athletes were recruited from the Singapore Sports School Track and Field Academy, Singapore. Participants were divided into two age groups: U19 (17–19 years; n = 8) and U16 (13–16 years; n = 10). These age groups corresponded to the ‘A’ Division (Junior College and Millennia Institute athletes, approximately 17–19 years old) and the ‘B’ and ‘C’ Divisions (secondary school athletes, approximately 12–16 years old), respectively. The boundary between these groups coincides with the transition from secondary to post-secondary education in Singapore. All participants were sprint specialists within their track and field programmes and were engaged in structured sprint-specific training five days per week at the time of testing. Participant characteristics are summarised in Table 1. Exclusion criteria included any musculoskeletal injury within the three months preceding data collection. Biological maturation was not directly assessed in this study (e.g., Maturity offset, Tanner stage); this represents a methodological limitation acknowledged in Section 4.5, as inter-individual variability in maturation status may influence neuromuscular characteristics independently of chronological age.

Written informed consent was obtained from all participants and, where applicable, from their legal guardians. The study was conducted in accordance with the Declaration of Helsinki and was approved by the Singapore Sport Institute Institutional Review Board, approval number: BM-EXP-046, issued date: 9 October 2023.

No a priori sample size calculation was conducted. The sample size was determined by practical constraints on participant availability: the number of youth track and field athletes able to take part in this multi-channel EMG protocol was limited, and it was not possible to balance group sizes because participants could not be reassigned across age categories. This resulted in an unequal group distribution (U19, n = 8; U16, n = 10). Post hoc observed power was not calculated, as it is a monotonic function of the obtained *p*-value and therefore provides no information beyond the significance test itself. In place of post hoc power, effect sizes are reported for every comparison: because the between-group comparisons of spatiotemporal variables involved independent samples, Cohen’s d is reported for each independent-sample *t*-test alongside and partial eta-squared (*η*^2^) is reported for all ANOVA effects. These estimates, together with their confidence intervals, allow readers to evaluate the magnitude and precision of the observed differences directly rather than inferring them from a post hoc power value. The constraints on statistical power arising from the small sample are acknowledged as a limitation in Section 4.5.

### 2.2. Experimental Design and Protocol

All sprint tests were performed on an outdoor synthetic straight sprint track covered by a roof, providing protection from direct sunlight and rain. Weather conditions were calm during testing. Following a standardised warm-up consisting of slow-jogging, dynamic lower-limb stretching (leg swings, hip circles, walking lunges), and progressive sprint build-ups at approximately 60%, 80%, and 90% of maximal effort over 30 m. Then, each participant performed 50 m sprint with maximal effort from a standing start. A recovery interval of at least 5 min was provided between trials. The trial yielding the highest maximum running speed was retained for analysis.

### 2.3. Spatiotemporal Variables

Spatiotemporal variables—running speed, step frequency, and step length—were measured over the 30–50 m section. A high-speed video camera (Lumix-Fz 300, Panasonic, Osaka, Japan) positioned perpendicular to the running lane was used to identify foot-strike and foot-off events (240 Hz), from which step frequency and step length were derived for five consecutive gait cycles within the 30–50 m measurement zone. The five cycles were selected as the first five complete cycles during 30–50 m. A dual-gate timing system (Brower Timing Gates System, Brower, UT, USA) computed mean running speed across the measurement zone.

### 2.4. Electromyography

**Figure 1:**
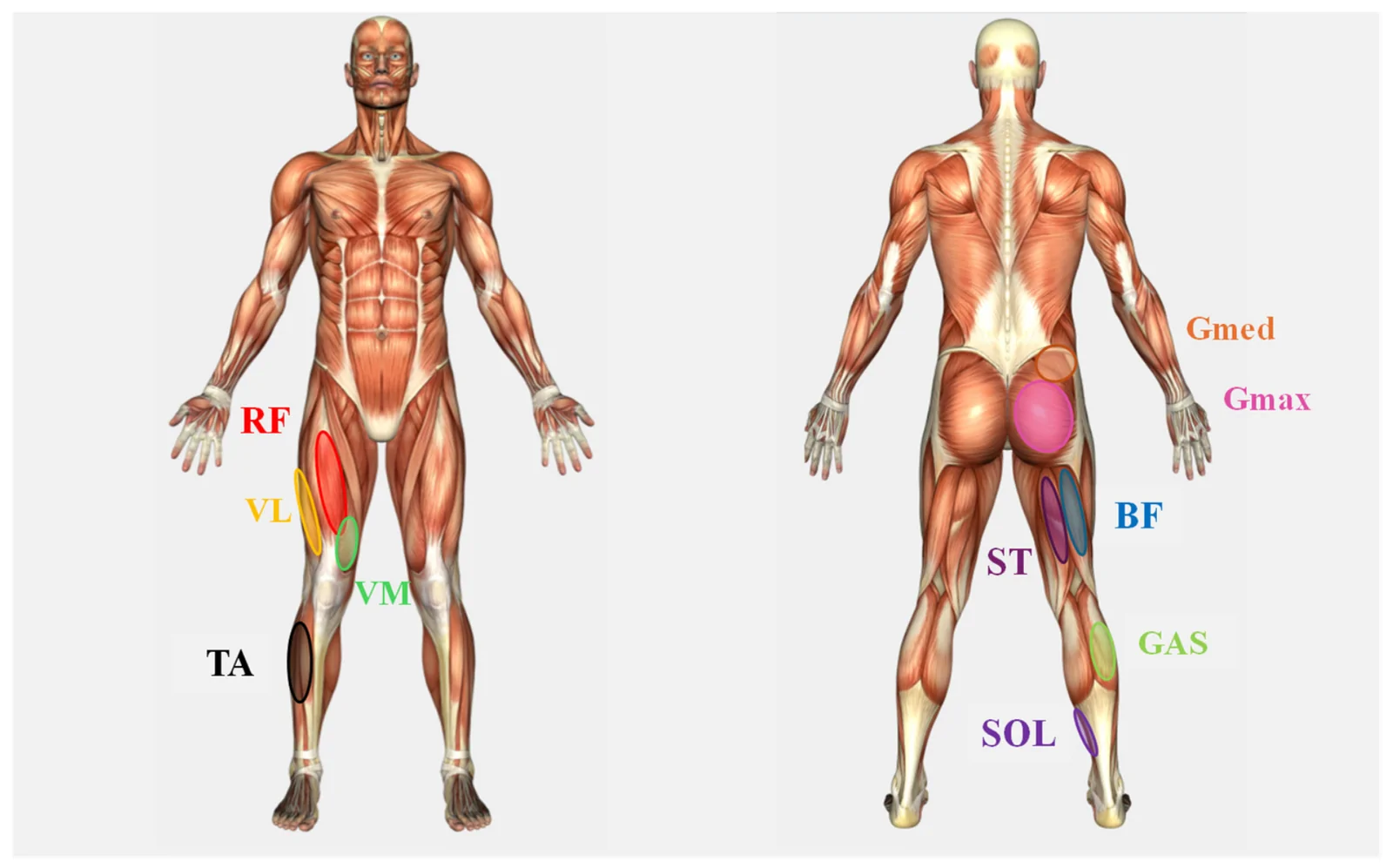
EMG sensor placement of ten tested lower limb muscles: rectus femoris (RF), biceps femoris long head (BF), semitendinosus (ST), gluteus maximus (Gmax), gluteus medius (Gmed), vastus lateralis (VL), vastus medialis (VM), tibialis anterior (TA), lateral gastrocnemius (GAS), and soleus (SOL).

Surface EMG signals were recorded simultaneously from ten lower limb muscles using wireless EMG sensors (Trigno Avanti Sensor, Delsys, MA, USA): rectus femoris (RF), biceps femoris long head (BF), semitendinosus (ST), gluteus maximus (Gmax), gluteus medius (Gmed), vastus lateralis (VL), vastus medialis (VM), tibialis anterior (TA), lateral gastrocnemius (GAS), and soleus (SOL) (Figure 1). Electrode placement followed SENIAM recommendations. Prior to placement, skin was shaved, abraded, and cleaned with isopropyl alcohol to reduce impedance.

Before the sprint trials, maximal voluntary isometric contractions (MVICs) were performed for each muscle, with standardised joint positions and stabilization procedures as follows:

- RF, VM, and VL: Knee extension, knee flexed 60°, hip flexed 90° (sitting position).
- BF and ST: Knee flexion, knee flexed 60° (sitting position).
- Gmax: Hip extension, knee flexed 90° (prone position).
- Gmed: Hip abduction, hip at 0°, knee fully extended (side-lying position).
- GAS and SOL: Ankle plantar flexion, ankle in neutral position, knee full extend, hip flexed 90° (sitting position).
- TA: Ankle dorsi flexion, ankle in neutral position; knee flexed 60°, hip flexed 90° (sitting position).

Three 5 s MVICs were performed, and the peak 1 s RMS from the highest-effort trial was used as the MVIC reference. EMG signals were band-pass filtered (20–450 Hz, fourth-order zero-lag Butterworth filter) and full-wave rectified. Root Mean Square (RMS) amplitude was computed across four discrete gait phases—contact, early-swing, mid-swing, and late-swing—defined from foot-strike and foot-off timings identified from the synchronised high-speed video. RMS values were normalised to respective MVIC values (% MVIC). This process was performed using MATLAB R2023b (MathWorks, Natick, MA, USA). Microsoft Excel and PowerPoint (Microsoft Corporation, Redmond, WA, USA) were also used for figure preparation.

### 2.5. Statistical Analysis

All analyses were performed using SPSS (version 28.0; IBM Corp., Armonk, NY, USA). Descriptive statistics are presented as mean ± SD. The Shapiro–Wilk test was used to assess normality for all variables. Homogeneity of variance was evaluated using Levene’s test for independent-sample comparisons. Sphericity for the within-subjects factor was assessed using Mauchly’s test; where violated, the Greenhouse–Geisser correction was applied. Between-group differences in spatiotemporal variables were assessed using independent-samples *t*-tests. For EMG data, a two-way mixed-model ANOVA was applied for each muscle, with group (U19 vs. U16) as the between-subjects factor and gait phase (contact, early-swing, mid-swing, late-swing) as the within-subjects factor. Separate ANOVAs were conducted for each of the ten muscles. No correction for multiple comparisons across muscles was applied, as each muscle was examined as an independent a priori research question based on its distinct anatomical and functional role; however, given that 10 tests were conducted, the possibility of inflated Type I error should be considered when interpreting the results, and the single significant finding should be interpreted with appropriate caution. Where a significant group × phase interaction was detected, post hoc pairwise comparisons with Bonferroni correction were applied. Effect sizes were calculated as partial eta-squared (*η*^2^) for ANOVA and Cohen’s d for pairwise comparisons, interpreted using conventional thresholds (*η*^2^: small = 0.01, medium = 0.06, large = 0.14; Cohen’s d: small = 0.2, medium = 0.5, large = 0.8). Given the small sample, effect sizes—rather than post hoc observed power—are used throughout to convey the magnitude and precision of the observed differences. Complete *t*-test results including *p*-values and Cohen’s d are reported in Table 1. Statistical significance was set at α = 0.05.

## 3. Results

### 3.1. Spatiotemporal Variables

The U19 group demonstrated significantly greater running speed (U19: 9.49 ± 0.39 vs. U16: 8.67 ± 0.25 m·s^−1^, *p* < 0.001), step frequency (U19: 4.49 ± 0.12 vs. U16: 4.35 ± 0.16 Hz, *p* = 0.004), and step length (U19: 2.12 ± 0.12 vs. U16: 1.99 ± 0.06, *p* = 0.010) than U16 (Table 1).

### 3.2. Lower Limb Muscle Activation

The overall pattern of lower limb muscle activation across the four gait phases was broadly comparable between the U19 and U16 groups for the majority of muscles. No statistically significant group × phase interactions were observed for BF, ST, Gmax, Gmed, VL, VM, TA, GAS, or SOL (all *p* > 0.05) as shown in Table 2, meaning that no significant between-group differences in phase-specific RMS amplitude were detected for these muscles. It should be noted, however, that the absence of statistical significance does not confirm equivalence of activation, and the study may have been insufficiently powered to detect smaller differences.

**Figure 2:**
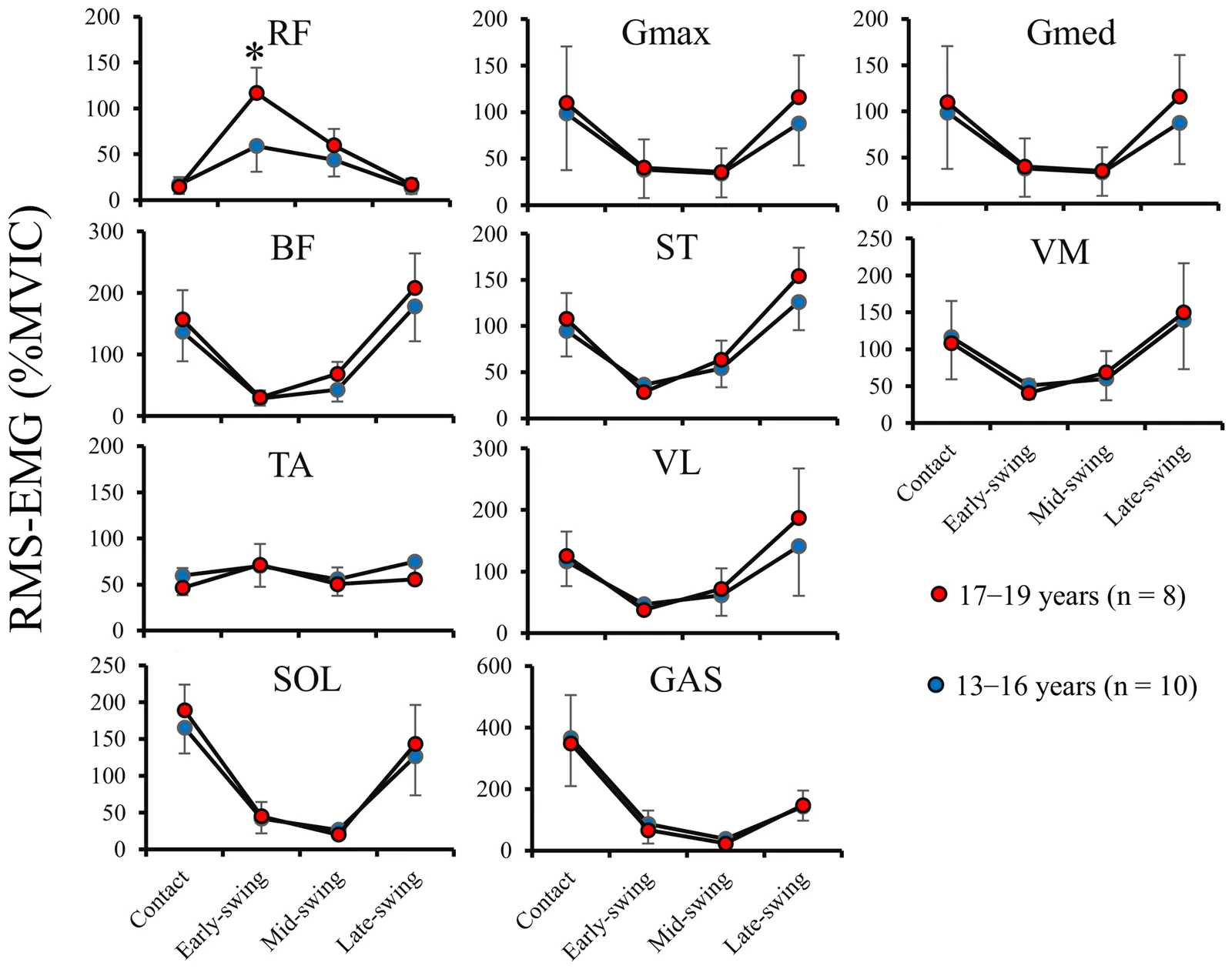
RMS-EMG activation (%MVIC) across four gait phases (contact, early-swing, mid-swing, late-swing phases) in U19 and U16 groups. *: RF activation during the early swing phase was significantly greater in U19 athletes.

A significant group × phase interaction was observed for RMS-EMG amplitude of RF (*F* = 5.257, *p* = 0.003, *η*^2^ = 0.247). Post hoc pairwise comparisons revealed that the U19 group exhibited significantly greater RF activation during the early-swing phase compared to U16 (*p* = 0.033). No significant between-group differences were observed for RF during the contact, mid-swing, or late-swing phases (all *p* > 0.05). These findings are illustrated in Figure 2. Error bars in Figure 2 represent standard deviations.

## 4. Discussion

The primary purpose of this study was to investigate whether neuromuscular activation strategies of the lower limb muscles during maximal sprinting differ between youth athletes of different age groups. Two key findings emerged. First, the overall pattern of lower limb muscle activation across the sprint gait cycle was broadly similar—in terms of the absence of statistically significant group × phase interactions—between U19 and U16 athletes for nine of the ten muscles examined, although this should not be interpreted as evidence of equivalence given the study’s limited statistical power. Second, RF activation during the early swing phase was significantly greater in U19 athletes compared to U16 counterparts, despite no significant differences in the other nine muscles. Together, these results may suggest that age-related differences in neuromuscular activation patterns are not characterised by a wholesale reorganisation of lower limb muscle coordination, but may instead involve more targeted differences in hip flexor activation at a phase-critical juncture of the gait cycle.

### 4.1. Robustness of the Overall Lower Limb Activation Pattern Across Age Groups

A foundational observation of the present study is that no statistically significant group × phase interactions were detected for nine of the ten muscles, despite clear and significant differences in spatiotemporal variables between age groups. This pattern is consistent with—though does not confirm—a broader principle in locomotion research: the global temporal structure of lower limb muscle activation during running is a highly conserved motor programme [14,23]. Muscle synergy analyses of running have demonstrated that the neuromuscular modules underlying locomotion are shared across developmental groups from childhood through elite adulthood [24].

The absence of significant differences for most muscles in trained youth athletes may suggest that the general timing architecture for sprinting is broadly established by early-to-mid adolescence in this population. However, it is important to acknowledge that with a sample size of 18 participants, the study may have been underpowered to detect smaller but potentially meaningful between-group differences in muscle activation. The present findings should therefore be interpreted as suggestive rather than definitive evidence of preserved neuromuscular organisation.

### 4.2. Greater RF Activation in U19 Athletes During Early Swing: Biomechanical Significance

The central finding—significantly greater RF activation during early swing in U19 compared to U16 athletes—has direct biomechanical relevance to sprint performance. The RF is a biarticular muscle crossing both the hip and knee joints [16]. Although its precise functional contribution during early swing involves both hip flexion and eccentric knee control, its role in driving forward limb recovery makes it particularly relevant to step frequency regulation [16,25]. In this context, greater RF activation amplitude during early swing may be associated with greater impulsive force applied to forward leg swing, potentially contributing to the higher step frequency observed in U19 athletes.

This interpretation is supported by the step frequency data. Biomechanical modelling has confirmed that hip flexors, including RF, require greater force production during early swing when step rate is higher [25]. The finding by Kakehata, Goto [16] that higher step frequency at maximal velocity is associated with earlier and stronger RF activation relative to BF activity provides a mechanistic framework for interpreting the present finding, although the present study did not directly test the relationship between RF RMS and step frequency, and these mechanistic explanations are therefore based on prior literature rather than being directly demonstrated here. Hip torque capability has also been proposed as a mechanistic link between acceleration and maximum velocity sprint performance [26].

It is also noteworthy that RF co-contraction with BF has been inversely related to sprint velocity [16,27], and that adolescents have been reported to exhibit higher RF–BF co-contraction than adults during the contact and propulsive phases of maximal sprinting [20], suggesting that efficient RF recruitment is characterised not only by magnitude but also by appropriate temporal dissociation from antagonist activity. Notably, in that adult–adolescent comparison, group differences in co-contraction emerged in the contact and propulsive phases but not in any swing-related phase [20]. The present finding of a between-group difference in RF activation amplitude during early swing therefore appears to capture a dimension of neuromuscular difference that co-contraction indices do not resolve, suggesting that the two approaches are complementary rather than redundant. The present phase-averaged RMS analysis does not permit inference about co-contraction indices; future studies employing fine-grained temporal analyses of RF-BF coordination across youth maturity groups would be a valuable extension of the present findings.

### 4.3. Structural vs. Neural Determinants: Re-Examining the Role of Type II Motor Unit Recruitment

A key interpretive challenge is determining the relative contributions of structural maturation and accumulated developmental experience to the observed group difference in RF activation. The between-group difference was confined specifically to RF and only during the early swing phase, while nine other muscles showed no significant group effect. This pattern of selective, phase-specific elevation is difficult to attribute to a general size-driven amplitude increase alone, and may instead reflect a neural control difference specific to the functional demands of early swing in the sprint cycle.

One neurophysiologically plausible mechanism is a difference in recruitment of high-threshold, type II motor units. Dotan and colleagues have proposed that age-related performance gaps in explosive tasks may partly reflect a lesser ability in younger athletes to recruit high-threshold type II motor units [8,10,28], and this hypothesis is consistent with the present finding. However, it is critical to emphasise that surface EMG RMS amplitude does not directly measure motor unit type or recruitment threshold. Surface EMG RMS amplitude reflects the summed electrical activity of all active motor units within the electrode detection volume and is influenced simultaneously by the number of active motor units, their discharge rates, motor unit action potential amplitude, and several peripheral factors including subcutaneous tissue thickness and electrode-to-fibre distance [29,30]. The inference from greater RF RMS to greater type II motor unit recruitment must therefore be treated as a plausible hypothesis rather than a demonstrated fact. Confirming this interpretation would require high-density EMG decomposition or intramuscular needle EMG—methodologies that represent important directions for future research in this population.

This motor unit recruitment hypothesis is made more plausible by the known muscle fibre composition of the RF, which has a relatively high proportion of type II fibres compared to other lower limb muscles [31,32]. Its fast-twitch fibre predominance makes the RF particularly dependent on high-threshold motor unit recruitment for explosive tasks, which may explain why a between-group difference in EMG amplitude was observed in RF but not in more slow-twitch-dominant muscles such as SOL or GAS. Histological analyses have also indicated that the relative proportion of fast-twitch fibres increases through puberty into early adulthood [32,33], although this maturational change in fibre composition would similarly affect multiple muscles, and cannot solely explain the muscle- and phase-specific nature of the present finding.

It is also important to acknowledge that, across ten muscle ANOVAs, only one significant finding was observed. When conducting multiple statistical tests, the probability of at least one false positive increases—with ten tests at α = 0.05, the probability of at least one spurious significant result by chance is approximately 40%. Although each muscle was tested as an a priori independent hypothesis, the isolated nature of the significant finding means it should be interpreted with caution and treated as a hypothesis-generating rather than a definitive result.

Taken together, the available evidence is consistent with the interpretation that the observed RF activation difference may reflect age-related differences in neural control of hip flexor recruitment during early swing, potentially associated with both accumulated sprint training and biological maturation. However, the cross-sectional design of this study cannot distinguish the independent contributions of training history, biological maturation, and other developmental factors to the observed group differences. Future longitudinal studies are needed to address this fundamental limitation.

### 4.4. Implications for Sport-Specific Testing, Screening, and Talent Development

The current findings may have practical implications for the assessment of youth sprinters. Existing talent identification frameworks are multidimensional, integrating anthropometric, physical, technical, psychological, and maturational characteristics [17,18]. Within this multidimensional framework, neuromuscular activation assessed via surface EMG may offer a complementary dimension of information; however, the predictive validity of EMG-based assessment for talent identification has not been evaluated in the present study, and its practical application requires further longitudinal and prospective validation before it can be recommended as a screening tool.

From a practical standpoint, advances in wireless surface EMG technology now make multi-muscle data collection during field-based sprint trials increasingly feasible [22]. The present findings provide a preliminary basis for investigating RF activation during early swing as a potential research variable in youth sprint assessment, pending replication in larger samples and evaluation of its predictive value for long-term athletic development.

From a training perspective, the present observational findings are consistent with the idea that developing hip flexor neuromuscular capacity may be a relevant target in youth sprint development. Hip flexor training has been shown to improve sprint performance in several cohorts [34], and the relationship between hip flexion power and step frequency further supports the functional relevance of this quality [26]. However, it should be emphasised that no training intervention was conducted in the present study, and these training recommendations are presented as hypotheses requiring future experimental validation rather than as prescriptions directly supported by the present data.

Research suggests that the period spanning approximately 13–16 years represents a particularly sensitive phase for motor coordination development and neuromuscular training responsiveness in male youth athletes [35,36]. Monitoring the trajectory of RF activation development longitudinally—and in relation to biological maturation status—could help clarify the extent to which the age-group differences observed here reflect training adaptations, maturational processes, or their interaction.

### 4.5. Limitations and Future Directions

Several limitations should be acknowledged. First, the cross-sectional design prevents causal inference: the group differences in RF activation cannot be attributed to either age-related maturation or accumulated sprint training experience, or distinguished from other developmental factors. The observed differences may reflect a combination of maturation, training history, anthropometric differences, and other age-related developmental factors.

Second, biological maturity was not directly quantified. Participants ranged from 13 to 19 years of age—a period of substantial inter-individual variability in maturation status—and chronological age alone cannot adequately represent neuromuscular developmental status. Future studies should incorporate maturity assessment (e.g., maturity offset, Tanner staging) to disentangle the independent effects of biological maturation and training history on RF activation.

Third, participant training characteristics were incompletely described. Detailed information regarding years of sprint-specific training, weekly training volume, and competitive level was not systematically collected, limiting the ability to interpret the findings in terms of training-related neuromuscular adaptation.

Fourth, no a priori sample size calculation was conducted, and the relatively small and unequal sample (U19, n = 8; U16, n = 10; N = 18) limits statistical power and generalisability. This reflected the practical reality of recruiting youth athletes able to complete a multi-channel EMG protocol during maximal sprinting, and the impossibility of balancing group sizes across fixed age categories. The absence of significant findings for nine muscles may therefore partly reflect insufficient power to detect smaller but meaningful differences rather than true neuromuscular equivalence. Replication in larger, independently recruited cohorts is warranted.

Fifth, ten separate ANOVAs were conducted without a correction for multiple comparisons. The single significant finding (RF) must be interpreted with caution, as it may represent a Type I error rather than a true neuromuscular difference.

Sixth, surface EMG RMS amplitude does not directly confirm specific neuromuscular adaptations such as type II motor unit recruitment. The mechanistic interpretation of the RF finding remains speculative and requires more in-depth neuromuscular assessment, including high-density EMG decomposition or intramuscular recording, to establish the motor unit basis of the observed group difference.

Finally, the study was restricted to male youth athletes; the extent to which findings generalise to female youth sprinters remains to be determined.

## 5. Conclusions

This study provides preliminary evidence that age-related differences in lower-limb neuromuscular activation during maximal sprinting in youth track and field athletes are not characterised by wholesale differences in muscle coordination, but may involve specific differences in hip flexor (RF) recruitment at the phase-critical moment of early swing. These findings extend existing neuromuscular assessment frameworks by introducing EMG-based evaluation of sprint-specific muscle activation as a potential complementary tool for youth athlete profiling—pending validation of its predictive value in longitudinal studies.

The overall lower limb muscle activation pattern across the sprint gait cycle showed no statistically significant group × phase interactions for nine of the ten muscles examined, a finding that should be interpreted cautiously given the study’s limited statistical power rather than as definitive evidence of neuromuscular equivalence. RF activation during the early swing phase was significantly greater in U19 athletes, a difference that is biomechanically consistent with faster leg recovery and higher step frequency. While the mechanistic basis of this finding—including potential differences in type II motor unit recruitment—cannot be confirmed from surface EMG alone, the phase-specific and muscle-specific nature of the group difference suggests it warrants further investigation with more direct neuromuscular assessment techniques.

Given the cross-sectional design, the observed RF activation difference cannot be attributed to sprint-specific training, biological maturation, or any other single developmental factor. Future longitudinal studies incorporating biological maturation assessment, detailed training history characterisation, and high-density EMG will be essential to establish the developmental trajectory of sprint-specific neuromuscular adaptations in youth athletes and to determine the applied potential of EMG-based assessment for talent identification and training prescription.

## Figures and Tables

**Table 1:** Participant characteristics and spatiotemporal variables (mean ± SD) for U19 and U16 groups, including independent-samples *t*-test results.

*Physical Characteristic* *and Athletic Level*	U19 Group (n = 8)	U16 Group (n = 10)	Independent *t*-test *p* Value	Effect Size Cohen’s *dz*
Height (m)	1.73 ± 0.06	1.66 ± 0.05	0.004 ^a^	1.48 ^b^
Body mass (kg)	64.2 ± 5.1	53.9 ± 7.7	0.002 ^a^	1.67 ^b^
Age (ys)	17.8 ± 1.3	13.9 ± 1.1	<0.001 ^a^	3.21 ^b^
WA score (pts)	802.8 ± 163.2	589.2 ± 112.8	0.003 ^a^	1.52 ^b^
***Spatiotemporal variables***				
Running speed (m·s^−1^)	9.49 ± 0.39	8.67 ± 0.25	<0.001 ^a^	2.58 ^b^
Step frequency (Hz)	4.49 ± 0.12	4.35 ± 0.16	0.004 ^a^	0.97
Step length (m)	2.12 ± 0.12	1.99 ± 0.06	0.010 ^a^	1.42 ^b^
Contact time (ms)	97.0 ± 5.5	100.8 ± 7.8	0.143	−0.56
Flight time (ms)	126.4 ± 6.3	129.4 ± 6.7	0.275	−0.46

^a^: Significant difference was observed (*p* < 0.05). ^b^: Detected effect size (*dz* > 1.36).

**Table 2:** Two-way mixed-model ANOVA results for the group × phase interaction in ten lower limb muscles RMS-EMG values during maximal sprinting.

Muscles	*F*-Value	*p*-Value	*η* ^2^
RF	5.257	0.003 ^a^	0.247 ^b^
Gmax	0.358	0.783	0.022
Gmed	0.358	0.783	0.022
BF	0.579	0.632	0.035
ST	1.859	0.149	0.104
VM	0.393	0.759	0.024
TA	2.878	0.051	0.139
VL	1.695	0.181	0.096
SOL	0.105	0.957	0.007
GAS	0.673	0.573	0.040

^a^: Significant intreraction was observed (*p* < 0.05). ^b^: Large effect size (*η*^2^ > 0.14).

## Data Availability

The data presented in this study are available on request from the corresponding author.

## Abbreviations

The following abbreviations are used in this manuscript:

EMG	Electromyography
RF	Rectus femoris
BF	Biceps femoris
ST	Semitendinosus
Gmax	Gluteus maximus
Gmed	Gluteus medius
VL	Vastus lateralis
VM	Vastus medialis
TA	Tibialis anterior
GAS	Gastrocnemius
SOL	Soleus
RMS	Root mean square
MVIC	Maximal voluntary Isometric contraction
